# Leadless Pacemakers: The “Leading Edge” of Quality of Life in Cardiac Electrophysiology

**DOI:** 10.1007/s11886-025-02228-5

**Published:** 2025-03-31

**Authors:** Samuel F. Sears, Elizabeth W. Jordan, Zeba Hashmath, Maeve M. Sargeant, John Catanzaro, Rajasekhar Nekkanti, Ghanshyam Shantha

**Affiliations:** 1 Department of Psychology, East Carolina University, Greenville, NC USA; 2 Department of Cardiovascular Sciences, East Carolina University, Greenville, NC USA; 3 Tulane University Medical Center, New Orleans, LA USA

**Keywords:** Pacemaker, Quality of life, Device acceptance, Patient management

## Abstract

**Purpose of the Review:**

Permanent pacemakers (PPMs) are common cardiac implantable devices indicated for patients with bradycardia or tachycardia. Currently, PPMs include both transvenous pacemakers (TV-PM) and leadless pacemakers (L-PM). This paper reviews the existing data on L-PM technology including: a) medical aspects and indications, b) patient experience and quality of life (QOL) outcome studies, and c) recommendations for optimizing patient QOL through enhanced knowledge and shared decision-making.

**Recent Findings:**

This review includes the seven papers that report on patient-reported outcomes (PRO) in leadless pacemakers and indicate that QOL is as good, if not better, than TV-PM. Existing evidence from descriptive statistics suggests that patients with L-PM report high levels of patient acceptance and satisfaction with esthetic appearance (96%), recovery (91%), and level of physical activity (74%).

**Summary:**

Leadless pacemakers provide an attractive alternative for the indicated potential patient. The evidence demonstrates the benefits of L-PM such as a minimal implant and lack of leads. Recommendations for future research indicate that electrophysiology-specific metrics are essential and control for the common co-morbidities in the PM population are needed.

## Introduction

The introduction of the pacemaker in the 1950s revolutionized the treatment of bradycardia and provided a “leading edge” of medical innovation in cardiology. Over the subsequent decades, innovation continued to refine the pacemaker's functions via lead technology to improve quality of life and remote management and minimize adverse effects via excessive RV pacing. Transvenous leads have remained a stubborn dimension of modern pacemakers due to complications with the pocket, infections, and lead failures.

Initially, single chamber leadless pacemakers were introduced and have now reported less infection and minimal technical complications out to a 3-year surveillance period, despite difficulty with achieving consistent atrioventricular synchrony resulting in them only being effective for about 20% of the patients who need pacemakers [1]. Knops et al. (2023) recently extended the leadless pacemaker revolution with dual chamber pacing and demonstrated suitable safety and efficacy data [2]. Recent state- of-the-art reviews highlighted the now 10 years of experiences with leadless pacemakers and their successes and significant future possibilities [3]. These authors point to the potential suitability of leadless pacemakers being paired with subcutaneous implantable cardioverter defibrillators and cardiac resynchronization therapies and even energy harvesting leadless pacemakers. Specifically, energy harvesting leadless pacemakers utilize energy generated by the heart’s natural movements or hemodynamics into electrical energy to power the device, with the goal of extending the lifespan of the pacemaker and reducing replacement surgeries. “Leading edge” developments like this strike interest and appeal to patients, but require systematic study and clinical trials. Moreover, contemporary research entails a commitment to understanding the patient experience through patient-reported outcomes (PRO), quality of life (QOL), and novel metrics. Employment of measures of the patient experience, such as patient acceptance, self-management, physical functioning, and quality of life, have not been routinely employed until more recently. The existing research provides some initial views of the patient experience to consider in both clinical and research applications.

This paper aims to review the existing data on leadless pacemaker (L-PM) technology on patient experience and quality of life (QOL) with comparison to transvenous pacemakers. The paper will review a) medical aspects and indications, b) QOL outcome studies and results, and c) postulate key areas of future investigation and recommend optimizing patient QOL through enhanced knowledge and shared decision-making.

## Existing Literature and Medical Aspects

### Medical

A wide range of patients with slow heart rates (bradycardia) benefit from PPM implantation. First are those with sinus node dysfunction: when the sino-atrial node (the natural pacemaker of the heart) that generates electricity in the heart, malfunctions and patients develop symptoms. Second are those with atrio-ventricular node dysfunction; i.e. patients who have defective electricity propagation between atrium and the ventricle. Finally, are those patients with atrial fibrillation, who either have slow heart rates or those who develop long pauses when they switch from atrial fibrillation to sinus rhythm.

Since the inaugural implantation of a pacemaker utilizing epicardial electrodes via thoracotomy by Senning in 1958, there has been a relentless pursuit of innovation aimed at refining the techniques of pacemaker implantation. These innovations have encompassed the progression from transcutaneous pacing to epicardial and transvenous pacemaker placement methods. Patients are conventionally managed with transvenous pacemakers (TV-PMs) and now approximate an annual implantation rate of about 1 million persons worldwide [4]. From an epidemiological perspective, this surge in implantation rates can be primarily ascribed to the aging population worldwide and the heightened survival rates among patients afflicted with heart-related ailments necessitating pacemaker intervention.

Traditional transvenous pacemaker implantation necessitates the creation of a subcutaneous pocket to house the pulse generator, in conjunction with venous access near the pericardial and pleural spaces (including the subclavian vein, axillary vein, or cephalic vein) for lead placement. Consequently, this procedure is susceptible to complications such as pocket hematoma, infection, lead perforation, pericardial effusion, pneumothorax, lead dislodgment, and hemorrhage [5–7]. Additionally, the long-term presence of a pacemaker may lead to further complications such as tricuspid valve damage, tricuspid insufficiency, and venous occlusion. Research has extensively examined the incidence rates of these complications, which typically range from 8 to 12% [5]. The post-implantation recovery period following TV-PM placement is invariably prolonged, with patients requiring a minimum of 4 to 6 weeks to resume their normal activities. This protracted recovery period is attributable primarily to pain at the implant site and restricted mobility in the arm on the side of pacemaker generator placement, which is essential to prevent lead dislodgment.

### Quality of Life in TV-PM Patients

The overall QOL benefit of the implantation of a pacemaker has been well-established for over two decades, with improvements in mental and physical quality of life across 11 studies [8–10]. The benefits of QOL are broadly sustained as well. Long-term generic quality of life data spanning a 7.5-year follow-up of 881 bradycardia pacemaker patients in the Netherlands indicated QOL scores remained improved from preimplantation over the study period. The absolute scores declined slightly but remained above preimplantation. This study is notable for using a pacemaker-specific QOL measure as well (e.g., Aquarrel Measure), which indicated similar benefits, except for an eventual decline in relief from dyspnea and decreased exertion at 5 years follow-up that was equivalent to pre-implantation levels [11]. Recent research and systematic reviews have confirmed patient benefits from TV-PMs [12, 13]. Collectively, pacemaker technology has produced perceptible QOL benefits for patients and warranted ongoing refinement and continued innovation.

### Past, Present, and Future of Leadless Pacemakers

The evolution of leadless pacemakers (LPs) has marked a significant shift in pacing technology. In 2012, the first leadless implanted pacemaker in patients was the Nanostim device {St. Jude Medical (now Abbott)}. Despite its pioneering status, the device was discontinued due to early battery depletion, prompting a technology redesign. In 2016 the Medtronic Micra transcatheter pacing system received the Food and Drug administration (FDA) approval and was introduced into clinical practice [14]. MICRA-VR, the single chamber right ventricular pacemaker capable of single chamber RV pacing, become the benchmark for leadless pacing, with over 150,000 implants worldwide providing robust data on safety and performance [15].

The era of dual chamber leadless pacing began with the FDA approval of MICRA-AV1 (the first-generation dual chamber leadless system from Medtronic) in January 2020. The mechanistic basis behind the need for dual chamber pacing is the essentiality of optimal atrio-ventricular (AV) synchrony for effective cardiac output. The ventricle should only contract when the atrium is relaxing and vice-versa, for optimal blood flow through the atrio-ventricular valves. Although an objective number for optimal AV synchrony is not established, patients are generally asymptomatic, when AV synchrony is above 70 – 75%. MICRA-AV1 utilized an accelerometer based mechanical sensing of atrial contractions. MICRA-AV1 could sense the atrium and pace the ventricle thereby showed promise for reasonable atrio-ventricular (AV) synchrony. The Marvel-2 study (Micra Atrial tRacking using a Ventricular accELerometer), showed 89.2% AV synchrony among its MICRA-AV1 study recipients [16]. However, in contrary, subsequent post approval studies with MICRA-AV1 reported much more conservative estimates of AV synchrony ranging between 33 – 80% [17–19]. Medtronic, then developed their second generation MICRA-AV2 that received FDA approval in May 2023. MICRA-AV2, in addition to enhancements in design for easier implantation, also had better programming capabilities. With optimal programming, MICRA-AV2 is capable to achieving, 90% AV synchrony in a wide range of patients with varying heart rates [16].

After the setback with Nanostim, Abbott came back with AVEIR-VR LP (Aveir leadless pacing), and received FDA approval after demonstrating favorable safety and efficacy in the LEADLESS II-phase 2 trial. In a recent study, Shantha et al. (2023) compared 25 patients who received the AVEIR-VR with an age- and sex-matched cohort implanted with the MICRA-VR device, reporting comparable effectiveness and safety between the two systems [20].

### Clinical Outcomes of Leadless Pacemakers

Research into dual-chamber leadless pacing has also yielded promising results. Knops (2023) reported that 90.3% of patients experienced freedom from complications at 90 days, with 90.2% achieving adequate atrial capture thresholds and sensing amplitudes at 3 months. Additionally, 97.3% of patients demonstrated at least 70% atrioventricular synchrony while sitting [2]. Most recently, 1-year data for the Aveir DR dual-chamber system showed an 88.6% freedom from system-related complications (95% CI: 84.5%–91.8%), a composite success rate of 92.8% for atrial capture and sensing thresholds (95% CI: 89.7%–95.8%), and a 90% success rate for communication between atrial and ventricular implants [21].

Leadless pacing has emerged as an effective alternative for patients with unique needs, such as those with limited vascular access (e.g., hemodialysis patients), those at high risk of infection, and younger patients aiming to avoid transvenous systems [15]. Current indications for LPs include high-grade AV block (paroxysmal or permanent) with or without atrial fibrillation and sinus node dysfunction. LPs are particularly valuable when dual-chamber transvenous pacing is deemed high-risk, complicated, or unnecessary.

Multiple clinical trials and meta-analyses have confirmed the benefits of LPs, including a lower risk of complications and excellent electrical performance. Ngo et al. (2021) reported a low complication incidence for Micra LPs (0.46% at 90 days and 1.77% at 1 year) and acceptable capture thresholds in > 90% of devices [20]. Notably, Micra LPs were associated with a 51% reduction in complications compared to transvenous pacemakers [22]. The latest 5-year data published in April 2024 further support these findings, demonstrating low rates of major complications (4.5% [95% CI: 3.6%–5.5%]) and system revisions (4.9% [95% CI: 3.9%–6.1%]) [23].

Wu et al. (2023) highlighted the safety and efficacy of LPs with atrioventricular synchrony algorithms, reporting a 6.3% overall complication rate, with significant complications related to the algorithm being rare. The pooled atrioventricular synchrony proportion was 78.9% [24]. The Aveir dual chamber leadless pacing system has been designed for AV synchronous pacing using wireless, beat-to-beat, implant-to-implant (i2i™) communication between distinct atrial and ventricular leadless pacemakers. Alhuarrat et al. (2023) reviewed national inpatient data from 2016–2019. They found higher in-hospital mortality and complication rates following LP implantation compared to single-chamber TVPs, likely due to more significant comorbidities in LP recipients [25]. Additionally, LPs offer comparable, if not superior, battery life to TVPs and are currently being studied for their retrievability.

### Pacing Induced Cardiomyopathy and Leadless Pacemakers

Pacing induced cardiomyopathy (PICM) remains the Achilles heel of RV pacing with a higher incidence noted with a higher burden of RV pacing [26]. Transvenous pacing is associated with a high incidence of PICM (14 – 36%) [26–28]. In contrary, leadless pacing is associated with a lower incidence of pacing cardiomyopathy compared to transvenous pacing. Sanchez et. al., in their observational study, compared 131 patients with TVP to 67 patients with LP. Incidence of PICM in their LP group (3%) was lower than their TVP group (13.7%) [29]. Further, Shantha et al. in their study of 358 patients with leadless pacemakers, reported a 7.8% incidence of PICM with 3 years follow-up in their total cohort [20]. In a subgroup analysis, the authors noted that the incidence of PICM was lower among those who received a high septal LP implant (4%) compared to a low apical septal implant (16.5%) [20]. This is in contrast to transvenous literature, where a high septal transvenous lead implantation did not lower incidence of PICM [28]. Although the mechanistic basis behind the discrepant associations of high septal LP implantations vs high septal implantation of TVPs with regards to PICM is unclear, these findings allude to the possibility of involvement of conduction system by high septal LPs. There is ongoing research assessing this possibility to develop future prototypes of LPs that can consistently perform conduction system pacing. Considering all the above data, the safety and efficacy of leadless pacemakers offer a promising alternative in cardiac pacing, offering innovative solutions for diverse patient populations.

## Patient-Reported Outcomes and Quality of Life in Patients with Leadless Pacemakers

The clinical scenario most associated with pacemaker consideration involves an older patient who is managing bradycardia, multiple comorbidities, reduced exercise tolerance, and hope to feel better. Therefore, baseline functioning must be considered when assessing the PROs or quality of life (QOL) response to pacemaker implantation. Research into Medicare beneficiaries in the United States suggests that approximately 75% of older adults regularly experience at least one of the symptoms of pain, fatigue, breathing difficulties, sleep difficulties, depression, or anxiety. Thirteen-point six percent had four or more of these symptoms [30]. An observational study of 119 Italian pacemaker patients indicated that 71% scored in the frail range and 71% had reduced activity scores at 12-month follow-up [31]. These data suggest that the value proposition of pacemaker implantation may have some limitations regarding the potential for improvements from a single modality intervention, such as a pacemaker, given competing causes of morbidity, symptoms, and dysfunction. Moreover, the process of TV-PM implantation requires activity restriction for 4 to 6 weeks to avoid complications and allow for lead endothelization. Conversely, the leadless pacemaker only requires 3 to 7 days of activity restriction [32]. Beyond the noticeable time differences, this scenario and expectation of activity may decrease patients’ fears about adverse events following their procedure.

Empirical evidence for QOL outcomes with the L-PM is based primarily on observational studies. The first was a single-arm observational study using a generic QOL metric and 720 leadless pacemaker patients. Results indicated that all domains of the SF-36 instrument improved from baseline to 3 months and 12 months. Importantly, L-PM patients reportedly were satisfied with the scars' esthetic appearance (96%), recovery (91%), and level of physical activity (74%) [33]. Similarly, Tjong et al., 2018 compared 106 non-randomized pacemaker patients (L-PM = 42: TV-PM = 64) and found that the L-PM patients reported better physical functioning, physical role functioning, and mental health than TV-PM patients [7]. Moreover, L-PM patients reported less discomfort, less restrictions, and less preoccupation about their health. Because this was not a randomized trial and used only PROs, these data can only be used for descriptive purposes, but remain supportive of potential advantages in terms of the patient experience [32].

The most robust comparison study between patients with L-PM and TV-PMs used propensity matching with 243 patients (77 pairs of L-PM & TV-PM patients) on both generic and device-specific QOL (e.g., Florida Patient Acceptance Survey) [34]. Researchers compared baseline, 1-week, 3-month, and 12-month QOL reports with key potential differences. Results indicated L-PM has less intra & post-op pain intensity, shorter hospitalizations, better device acceptance, and better mental & physical QOL scores. Notably, the procedure time was longer for the leadless implantation procedure (42 vs. 28 min) [34]. This may indicate that individuals with L-PMs report improved health outcomes due to having fewer concerns or psychological distress about their device. Consequently, this experience can make them feel more encouraged and safer to engage in physical activity, thus improving their overall outcomes after device implantation.

Most recently, Yu et al. (2023) reported more significant improvements in L-PM vs.TV-PM patients in metrics of physical function, mental health, and social functioning [35]. These findings emphasize that patients with the leadless pacemaker can feel normalcy despite living with a cardiac device, which can, in turn, increase their overall health outcomes. Finally, Chinitz et al., 2023 reported that patients in a prospective single-arm trial reported improved generic QOL from pre-implant to 3-month follow-up [19]. Table 1 provides a full roster of studies on QOL. While still observational, these data provide us with the broadest view of the patient experience for comparison of the pacemaker types.

**Table 1:** Quality of life research summary

Paper	Purpose	Methods	Results
Piccini, et al., 2021 [38]	Compare patient characteristics and complications among patients implanted with leadless VVI and transvenous VVI pacemakers	• *N* = 5,746 L-PM • *N* = 9,662 TV-PM • Propensity score overlap • Observational study using insurance claims database	• Less complications in L-PM vs. TV-PM through 6 months • Pericardial effusion or perforation was significantly higher in L-PM vs. TV-PM within 30 days • No difference in acute complications after adjusting for patient characteristics
Bodin, et al., 2022 [39]	Assess and compare real‐life clinical outcomes within the first 30 days and during a midterm follow‐up with the 2 techniques	• *N* = 1,487 L-PM • *N* = 40,828 TV-PM • *N* = 1,344 Propensity Matched Pairs • Longitudinal study using hospital database	• Lower all-cause and cardiovascular mortality in L-PM within 30 days • No significant difference in all-cause death, cardiovascular death, and infective endocarditis between L-PM and TV-PM after matching for all baseline characteristics at midterm follow-up
Tjong, et al., 2018 [7]	Provide a balanced comparison of leadless and transvenous single-chamber PM therapies through a propensity score-matched analysis	• *N* = 254 L-PM • *N* = 281 TV-PM • *N* = 220 Propensity Matched pairs • Retrospective study	• Excluding PM complications, complication rate at 800 days was 0.9% for L-PM and 4.7% for TV-PM • Including PM complications, complication rate at 800 days was 10.9% for L-PM and 4.7% for TV-PM
Palmisano et al., 2021 [34]	Compare the L-PM to TV-PM on medical resources, patient comfort, and therapy acceptance	• *N* = 77 Propensity Matched pairs • Prospective study	• Implantation procedure was longer in L-PM than TV-PM • L-PM had lower intra- and post-operative pain intensity, shorter hospitalization, greater patient acceptance, and better QOL on both physical and mental health scales
Cabanas-Grandío, et al., 2020 [32]	Compare quality of life between patients with L-PM and C-PM	• *N* = 42 L-PM • *N* = 64 C-PM • Observational study	• No difference in baseline QOL • L-PM had higher QOL in both physical and mental health at 6-months • L-PM reported significantly higher physical function, physical role, and mental health • L-PM reported significantly less pacemaker-related discomfort and physical restrictions
Yu, et al., 2023 [35]	Compare the quality of life in Chinese patients undergoing leadless versus conventional pacemaker implantation	• *N* = 35 L-PM • *N* = 84 C-PM • Observational study	• No difference in baseline QOL scores between groups • L-PM were significantly better at physical function, physical role, bodily pain, vitality, social function, emotional health, and mental health at 3-month follow-up • L-PM reported significantly lower pacemaker-related discomfort and mobility limitations
Chinitz et al., 2023 [19]	Determine the performance of a leadless ventricular pacemaker with accelerometer-based algorithms for AVS pacing	• *N* = 152 L-PM • Prospective, single-arm study	• L-PM was associated with significant improvement in QOL from baseline to 3-month and sustained change to 6-months

Key: Leadless pacemaker (L-PM), transvenous pacemaker (TV-PM), quality of life (QOL), pacemaker (PM), conventional pacemaker (C-PM), atrioventricular synchronous pacing (AVS pacing)

## Clinical and Research Implications

### Clinical Implications Pre-Implant

Engaging patients in decision-making related to selecting cardiac pacemakers remains challenging. As an initial first step, breakthrough technologies must establish patient safety and clinical efficacy. Patients expect safety and effectiveness but may not know how to inquire thoroughly about QOL priorities. The challenge to innovators and industry involves creating sufficient evidence for the QOL factors, so that decision processes can go beyond the “wow factor” of the technology.

The initiation of the shared decision-making era has reduced the paternalism of medicine to a shared process in which different kinds of information are now needed to address patient priorities. Burri (2022) provided a table of pre-implant considerations to facilitate shared decision-making [36]. Patients want to be informed about the pros and cons of novel technologies to increase their device acceptance and certainty in their ability to live and achieve QOL goals with the device. Shared decision-making aspires to be fully informed, enhance patients’ confidence in engaging in activities, and improve their QOL post-implantation. In the future, increased data regarding clinical and patient reported outcomes will assist in comparing medical outcomes and advance shared decision making. Table 2 describes some key clinical considerations to facilitate shared decision-making with information relevant to pre- and post-implant considerations to optimize patient engagement and pursuit of QOL.

**Table 2:** Proposed clinical implications for leadless pacemaker discussions

Pre-Implant	Post-Implant
• Describe the pacing problem and potential device solutions • Engage in shared decision-making with the specifics of quality-of-life change potential • Allow for patient and family consultation about specifics • Discuss both short and long-term outcomes of device decisions • Receive the decision and confirm the rationale for the choice	• Acknowledge the potential value of technology • Empathize with limits of age and comorbidities • Encourage “testing the limits” related to physical activity • Recognize fears and potential gains • Target Movement: Early, Often and Always • Activate family to pursue quality-of-life activities

### Clinical Implications Post-Implant

Following a successful shared decision-making process, the hope is that patients will feel capable of returning to daily activities, and providers should encourage patients to increase physical activity as soon as possible. Given the spectrum of patients that receive these technologies, it is essential to address and empathize with limits to activity, given factors such as age and comorbidities. However, physical activity can be tailored to the patient, and resources such as cardiac rehabilitation can be utilized. Returning to activity and reducing pain or discomfort in the first month of recovery appears critical. Research indicates that approximately 12% of pacemaker patients remain on opioid prescriptions for over a month following the implantation of cardiac devices [37]. Lastly, family and partners should be aware of the patients’ needs to increase activity engagement to help promote these behaviors and facilitate improved quality of life.

### Research Implications

**Fig. 1:**
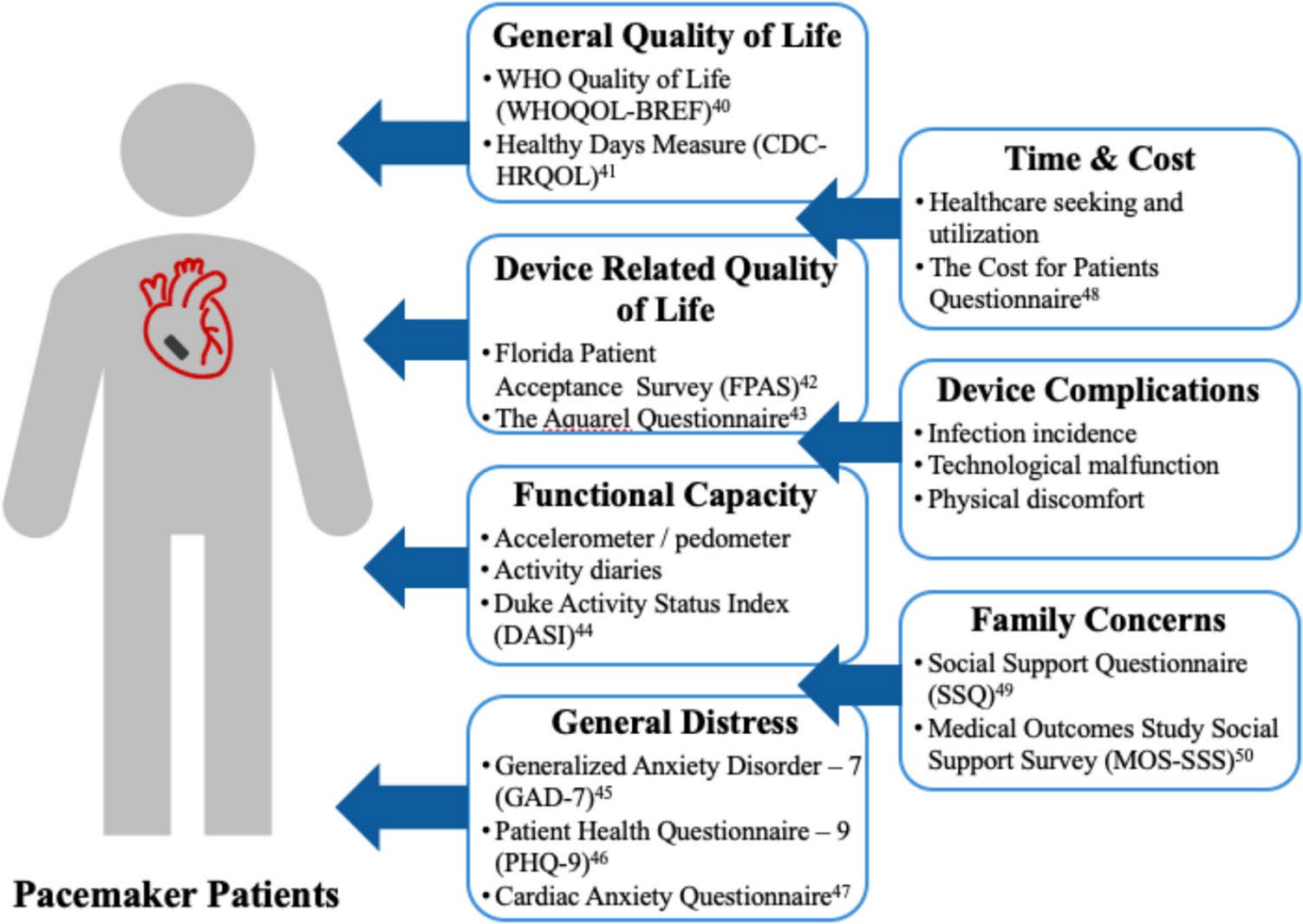
Potential scope of relevant patient-reported outcomes and suggested surveys [40–50]

Current evidence indicates that patients generally have positive experiences with leadless pacemakers. However, existing studies face notable limitations, including non-randomized designs and simplistic approaches to measuring patient experiences. Most rely on generic QOL tools, such as the SF-36 and EQ-5D at 30 days. These metrics are useful for comparing pacemaker patients with same-age peers without pacemakers but may fail to portray the specific benefits of pacing. This is due to the confounding effects of comorbidities and other conditions that influence quality of life and create noise of measurement. Device-specific and disease-specific quality-of-life tools offer a more precise assessment of the benefits of pacemaker therapy. Among the studies reviewed, Palmisano’s research is the only one that incorporates device-specific metrics [34]. Figure 1 provides an analysis of possible PROs and relevant questionnaires for the leadless pacemaker studies. A key challenge is the absence of randomized controlled trials (RCTs) comparing leadless to transvenous pacemakers. While RCTs would provide more substantial evidence of the relative benefits between the two devices, their feasibility is hindered by high costs and the difficulty of recruiting patients willing to be randomized. Although this may set an unreasonable standard, it leaves questions about the comparative patient experience between the two approaches. Addressing these research gaps is essential to enhance the shared decision-making process, allowing patients, families, and clinicians to make more informed and personalized treatment decisions.

## Conclusions

Leadless pacemaker systems represent the “leading edge” of cardiac electrophysiology and the future due to their technical elegance and potential patient benefit. The existing research suggests that patients perceive substantial benefit from leadless pacemakers, and the ability to return to activity is one predominant factor. Innovators have attempted to overcome the limits of the transvenous pacemaker with its pocket and potential infections, but leadless pacemakers also allow for a quicker return to activity. Clinical and research use of the leadless pacemaker suggests that comorbidities play a more significant role in this population, likely due to frailty and multiple potentially disabling conditions. Multidisciplinary teams that can address key aspects of accepting this technology, re-engaging in daily and physical activities, and returning to a whole life are indicated. Continued innovation using leadless approaches will likely continue to provide evolution in cardiac electrophysiology.

## Key References

- Crossley GH, Piccini JP, Longacre C, Higuera L, Stromberg K, El-Chami MF. Leadless versus transvenous single-chamber ventricular pacemakers: 3 year follow-up of the Micra CED study. J Cardiovasc Electrophysiol. 2023:34(4):1015–1023. 10.1111/jce.15863
  - ○ Findings from this study suggest that leadless pacemakers were associated with decreased complications, heart failure hospitalizations, and infections.
- Yu M, Li YP, Shi DM, Zhou YJ. Comparation of quality of life in Chinese patients undergoing leadless versus conventional pacemaker implantation. Clin Cardiol. 2023:46(1):49–56. 10.1002/clc.23939
  - ○ Findings from this study suggest that patients with leadless pacemakers had increased physical and social function, emotional health, and mental health at 3 months post-implant.

## Data Availability

No datasets were generated or analysed during the current study.
